# Are There Any Cognitive Benefits of Computer-Based Foreign Language Training for Healthy Elderly People? – A Mini-Review

**DOI:** 10.3389/fpsyg.2020.573287

**Published:** 2021-01-12

**Authors:** Blanka Klimova

**Affiliations:** Department of Applied Linguistics, Faculty of Informatics and Management, University of Hradec Kralove, Rokitanskeho, Czechia

**Keywords:** cognitive functions, seniors, foreign language learning, computer-based training, benefits

## Abstract

The purpose of this mini-review is to investigate if there are any cognitive benefits of computer-based foreign language training for healthy older individuals aged 55+ years. The author conducted a literature search of peer-reviewed English written research articles found in Pub Med, Web of Science and Scopus. The findings of this mini-review reveal that the research on the cognitive benefits of computer-based foreign language training for healthy older individuals is small-scale. The limited research findings of only three relevant studies indicate that these computer-based foreign language training programs may bring cognitive benefits for healthy elderly people, especially as far as the enhancement of their cognitive functions such as working memory are concerned. In addition, the authors of these studies suggest that foreign language learning is a useful activity for healthy older adults since it has the benefits of being meaningful (an advantage over other cognitive training approaches) and provides the chance for acquiring important skills that can benefit other aspects of life, such as travel or communication. In conclusion, the author of this mini-review also provides several implications for practice and future research.

## Introduction

At present the population is rapidly aging. The statistics shows that in 2015 there were about 900 million people aged 60+ years, and in 2050 this number is estimated to reach 2 billion (WHO, 2020a). By 2020, the number of people aged 60+ years will outnumber children younger than 5 years (WHO, 2020a). This presents many challenges to society, such as increased demands on healthcare systems, social security, or pension schemes. Generally, this aging trend represents a significant economic and social burden for the entire society (Klimova et al., 2016a).

Aging brings serious changes to an individual, such as deterioration of his/her physical, mental, and cognitive abilities (Charles and Carstensen, 2010). The most significant changes are declines in cognitive abilities which demand fast processing of information or to making quick decisions (Murman, 2015). Particularly, the fluid intelligence, i.e., the ability to reason and solve things, is impacted in older age (Kurdziel et al., 2017). These changes are part of the normal aging process. Research (Mazzonna and Peracchi, 2012) shows that especially after retirement the age-related cognitive decline increases because after retirement, individuals lose the market incentive to invest in cognitive repair activities. Therefore, more attention should be paid to extending active life of healthy older people. The reason is that more serious difficulties with memory, language, thinking, or judgment may signify an age-related cognitive disorder, such as dementia.

Currently, there are about 50 million people living with dementia, and by 2050 this number is projected to triple, i.e., there will be about 150 million people suffering from dementia (WHO, 2020b), which will represent 7.5% of the worldwide population. As research (Christensen, 2001) also reveals, a diversity in cognitive aging exists, which suggests that more than one process may be operating to produce the observed cognitive outcomes. As Christensen (2001) states, education, good health, absence of the APOE epsilon4 allele, and activity may be protective of cognitive decline. Furthermore, Park and Reuter-Lorenz (2009) proposed the scaffolding theory of aging and cognition (STAC), an integrative view of the aging mind, suggesting that pervasive increased frontal activation with age is a marker of an adaptive brain that engages in compensatory scaffolding in response to the challenges posed by declining neural structures and function. In addition, differences in genetic and lifestyle factors may also affect brain maintenance in aging. For example, lifestyle factors such as engagement in stimulating leisure activities may influence cognitive performance by preserving the brain’s gray and white matter integrity (Nyberg et al., 2012). This has been supported by other studies, e.g., Drigas et al. (2018), Klimova and Kuca (2015), Li et al. (2017), or Rosenberg et al. (2020), who report that non-pharmacological activities which are aimed at the stimulation of the cognitive functions might delay the cognitive decline. In this respect, clear evidence has been confirmed by studies on physical activities (Langhammer et al., 2018; Esmail et al., 2019), cognitive training (Borella et al., 2013; Rizkalla, 2015), as well as healthy dietary patterns (Solfrizzi et al., 2018; Soldevila-Domenech et al., 2019). But other strategies are emerging, such as foreign language learning (Bubbico et al., 2019; Wong et al., 2019) or computerized cognitive training (Corbett et al., 2015; Klimova, 2016), which might help enhance cognitive performance of older people. However, the evidence of these two latter strategies is not convincing. For instance, Berggren et al. (2018) in their study with 160 healthy older participants aged between 62 and 75 years, divided these subjects into an experimental group who studied Italian twice a week for 2.5 h for 11 weeks, and a control group who did relaxation training once per week for 11 weeks. No evidence of cognitive improvement through foreign language learning (FLL) was found. The same was true for Ramos et al. (2017) in their study with older Spanish people. They discovered that the switching ability (i.e., the ability to shift attention between one task and another) was not enhanced by FLL, in this case Basque language. In their review study Gates et al. (2020) found low-quality evidence suggesting that immediately after completion of the intervention small benefits of CCT may be seen for global cognitive function when compared with active controls, and for episodic memory when compared with an inactive control. Similarly, research findings by Owen et al. (2010) show that there is not much evidence for the improvement of general cognitive functions in the wider population through computerized cognitive training.

Thus, the purpose of this mini-review is to investigate if there are any cognitive benefits of computer-based foreign language training for healthy older individuals at the age of 55+ years.

## Foreign Language Learning and Older Individuals

As Antoniou et al. (2013) report, FLL in later age seems to be a quite important activity since it may enhance cognitive reserve of older adults. FLL involves a huge brain network that is known to coincide with the areas negatively influenced by the aging process (Murman, 2015). The brain plasticity helps maintain cognitive performance in the face of structural decline. The hypothesis that FLL may delay cognitive decline is supported by studies on bilingualism (Antoniou et al., 2013; Bialystok, 2017; Antoniou, 2019), which indicate that cognitive decline in later life among healthy bilingual elderly may be delayed by a few years (Bialystok et al., 2007; Craik et al., 2010; Bak et al., 2014). Furthermore, Antoniou and Wright (2017) suggested certain mechanisms which contribute to brain advantages affected by FLL. These are, for example, language typology, age of acquisition or bilingualism versus multilingualism. Nevertheless, there are research studies (cf. Paap et al., 2015), which deny the evidence of bilingual advantages on general executive functioning.

Klimova and Pikhart (2020) in their review on the effect of FLL on cognitive functions among healthy elderly showed that cognitive performance might be maintained and in some cases even improved (cf. Pfenninger and Polz, 2018; Bubbico et al., 2019; Wong et al., 2019), although two of the reviewed studies (Ware et al., 2017; Valis et al., 2019) did not detect any cognitive improvement through FLL. In addition, the authors indicate that these results should be critically evaluated since there were differences in methodologies, e.g., some lacked follow-up periods or control groups, and the length of the interventions varied.

Although, in comparison with younger adults, seniors are not usually that fast in improving their linguistic achievement (Gabrys-Barker, 2017) because their cognitive functions (e.g., processing speed, working memory, attention, or inhibitory control) decline with advancing age and they also suffer from a loss of sensory acuity (Singleton and Pfenninger, 2018), they are highly motivated to learn a foreign language. FLL offers them a new purpose of life (Pot et al., 2019), social inclusion (Narushima et al., 2018), increased self-efficacy (Chu and Chu, 2010), reduces feelings of anxiety and depression (MacIntyre and Dewaele, 2014; Pot et al., 2017), or evokes in them the feelings of happiness, joy, and satisfaction (Dewaele et al., 2019). For these reasons, FLL seems to be highly beneficial to the overall wellbeing of older people (Pot et al., 2017).

## Computerized Cognitive Training and Older Individuals

Technologies have now become part of seniors’ lives. The statistical data show that 70% of older adults now go online (WEF, 2019) and two thirds of them at the age of 65–69 years use the high-speed Internet at home (Pew Research Center, 2017). This number is expected to grow since present active seniors (i.e., still working people) at the age of 60 years use the Internet on a daily basis (Klimova et al., 2016b). The data also reveal that Internet use in those aged between 65 and 74 years increased from 52% in 2011 to 78% in 2017 (Lifeline24, 2019).

Older people use computers for communicating with their relatives and friends, making appointments with doctors, shopping, developing leisure and entertainment opportunities, or life-long learning habits (Gonzales et al., 2015; Klimova and Poulova, 2018). In this way, technologies enable them to stay independent and socially inclusive. Being connected enables older people to prevent their loneliness and social isolation (Fan, 2016; Liyanagunawardena and Williams, 2016).

In addition, technologies seem to play an important role in cognitive training. For example, Kueider et al., 2012 explored the efficacy of computer-based cognitive interventions for cognitively healthy older adults, with a special focus on cognitive outcomes as a result of training. Their findings are comparable or better than those from reviews of more traditional, paper-and-pencil cognitive training approaches suggesting that computerized training is an effective, less labor intensive alternative. But, as Butler et al. (2018) claim, cognitive training especially improves cognitive performance in the domain trained (cf. Butler et al., 2018). Several computer-based cognitive training programs exist which can improve older people’s reasoning skills, short-term memory, working memory, processing speed, and visual working memory (McAvinue et al., 2013; Corbett et al., 2015; Walton et al., 2015). Klimova (2016) in her review study showed that computerized cognitive training may lead to the improvement of cognitive functions, such as working memory and reasoning skills in particular. This training should be performed over a longer time span since a short-term cognitive training mainly has an impact on short-term memory with temporary effects (McAvinue et al., 2013; Walton et al., 2015). In addition, the training must be intense to be effective (cf. Zelinski et al., 2011; Haesner et al., 2015).

Research performed both on FLL and computerized cognitive training also suggested certain methodological recommendations with respect to the fact that older people form quite a heterogeneous group of learners with specific needs which should be individually addressed. For example, tutors should consider the learning environment, which should bring together people with the same level of acquired knowledge in order to avoid the feelings of lower self-confidence and anxiety if they saw that their peers are faster and better at learning new things. Furthermore, Pappas et al. (2019) report that especially e-learning courses for older adults should be short, clear, logical, and comprehensive; educational materials should be broken in small units to keep their attention and the teachers should provide constant feedback to this group of people to help them understand both the learning materials and their progress, as well as give them enough time to absorb new knowledge. In addition, the topic they study must be appealing to them enough to make them engaged in their learning. This has been supported by Ordonez et al. (2017), but also by Liyanagunawardena and Williams (2016) in their study on elderly learners and massive open online courses. Their findings indicate that most of the seniors participated in a health-related course titled *Heart Health*.

## Methods

The methodology of this mini-review is based on a literature search of peer-reviewed English written research articles found in Pub Med, Web of Science, and Scopus. The articles were queried for the following keywords: *second language learning* AND *elderly, second language learning* AND *elderly* AND *technologies, technologies* AND *older people* AND *foreign language learning*, *technologies* AND *elderly* AND *foreign language learning, online language courses* AND *elderly, online language courses* AND *older people, computer-based language programs* AND *elderly*. The studies had to include healthy older individuals at the age of 55+ years who studied a foreign language using a computer. The search was not limited by any time period. The author also conducted a backward search, i.e., she searched the references of detected studies for relevant research studies which could be missed during her research. In addition, a Google search was performed in order to detect unpublished (gray) literature, such was a case of Master’s Thesis by van den Berg (2019).

## Computer-Based Foreign Language Training for Healthy Individuals

Generally, empirical studies on this research topic are very sparse. The author detected only three empirical research studies: a Dutch pilot study by van den Berg (2019); a French pilot study by Ware et al. (2017); and Wong et al.’s study (2019), which is a Chinese study. The main topic was learning a foreign language, especially retention, by older healthy individuals and the use of technologies. In two cases, older adults were learning English and in one Spanish. The studies used standardized outcome measures, i.e., standardized tests for measuring cognitive functions, questionnaires, post-intervention, semi-directive interviews, or a content/theme analysis. However, methodologies of these studies differ significantly, which again might affect the reliability and validity of the research findings. The main limitations include a lack of control groups, small sample sizes, different outcome measures, and the length of intervention periods, which are all critical when assessing long-term changes in cognitive performance.

Ware et al. (2017) in their qualitative and exploratory study developed a technology-based English training program for 14 older French adults (nine females and five males). The program was based on the assumptions provided by Antoniou et al. (2013). These assumptions involved various factors, such as that computer-based language training can be administered anywhere and at any time to suit learner’s needs, the content can be adjusted and items can be repeated. In addition, learners can socialize. The average age of the participants was 75 years. The course lasted for four months and consisted of 16 two-hour sessions. The program was based on a multimedia approach using online videos and dictionaries on tablets or laptops. The participants were offered content from public-domain television series that aired in the 1950s and 1960s, as well as popular music by artists of the era, reviving memories from participants’ youth, which researchers thought they might find interesting. In addition, during each session, they studied one topic familiar to them, such as making breakfast, gardening, or shopping for clothes. Participants were also encouraged to train at home by connecting to the websites shown in class in addition to attending the group lessons. The participants found the program feasible, stimulating, and enjoyable, although some of them, who lacked previous experience with English, had difficulties with it. However, no gains on cognitive and mental performance were detected. The authors assume that this might have been caused by study’s small sample size, as well as participants’ generally high cognitive level. These results could also imply that the intervention played a role in the maintenance of participants’ cognitive level. Although research has focused on early bilingualism’s contribution to cognitive reserve, it has been shown that even those who acquire a language after childhood have a cognitive advantage over monolinguals. For instance, compared to their monolingual peers, late bilinguals have demonstrated higher scores in tests of auditory attention span. Thus, the authors believe that FLL can be a good cognitive intervention. Nevertheless, the results also showed the need for computer training and the importance of social ties which participants would appreciate.

Wong et al. (2019) in their randomized controlled study worked with 153 healthy seniors between the ages of 60 and 85 years from Hong Kong. The subjects were divided into three groups: (1) foreign language group, in which they learned basic English (experimental group); (2) games, in which subjects played cognitively stimulating activities, for example, puzzles (active control group); and (3) music appreciation group, in which subjects watched traditional and contemporary Chinese music videos (passive control group). All these intervention trainings lasted up to 5 h per week for 6 months. In the experimental group, subjects used a computer-based language training software called Rosetta Stone (2019), which helps learners speak English in conversations with bite-sized lessons that focus on delivering spoken words alongside visual and audio cues. The findings of this study reveal that computer-based foreign language learning and games, but not music appreciation, improved overall cognitive abilities that were maintained at 3 months after training. In addition, FLL had a positive impact on the enhancement of working memory, which may be connected with the beginning stages FLL, when a focus is often on vocabulary learning. Learners are presented with new words that are mapped onto pictures over many trials. This process requires working memory since learners must temporarily retain new words and immediately identify pictures with the new words. On the contrary, participants in the game group improved in attention, which might be associated with monitoring participants’ and players’ moves, as well as by suppressing irrelevant information while enhancing orientation to relevant information.

The improvement of working memory through FLL was also true for the participants of van den Berg’s study (2019). She conducted an experiment on improving cognition through FLL in seven older Dutch adults aged between 64 and 78 years who participated in a 10-day (1 h lesson per day) online Spanish course. Van den Berg also used Rosetta Stone software described above for FLL. The findings revealed no cognitive benefits in the third-age learners, which might have been caused by the small subject sample, the absence of a control group, a short intervention period, outside classroom settings if compared with Wong et al. (2019), or the language typology since Spanish is typologically distinct from Dutch. Although the participants boosted their working memory, they did not improve their attentional control and wellbeing. The key findings of this study show that older adults are able to quickly achieve vocabulary learning through an immersive online foreign language course.

The results of this mini-review illustrate that the research in this field is very scare, which might be associated with difficulties finding healthy older adults for an experiment. They are either too busy with many activities on their shoulders (Valis et al., 2019) or suffering from different diseases (van den Berg, 2019). Therefore, researchers are sometimes not able to form a control group. Furthermore, such research also requires a multidisciplinary team (a computer expert, a linguist, a psychologist, a statistician, as well as a foreign language teacher), which may be another problem. All these issues are also reflected in the limitations of the reviewed studies described above.

The only study which generated convincing cognitive benefits was that by Wong et al. (2019). The results of this study show that intensive FLL initiated in later life brings cognitive benefits. The authors also report that the more engaging an activity is, the more likely the cognitive improvement. The findings of those few examined studies also indicate that both short and longer intensive computer-based foreign language learning seems to bring cognitive benefits for healthy elderly people, especially enhancement of their working memory (van den Berg, 2019; Wong et al., 2019). This might confirm the statement by Butler et al. (2018) that cognitive training especially enhances cognitive performance in the domain trained. Ware et al. (2017) also confirm a cognitive advantage of bilinguals in FLL over monolinguals. They reveal that older individuals, particularly in early stages of learning, can achieve the same learning gains as their younger counterparts (van den Berg, 2019). They can build on their previous learning experience and linguistic knowledge. However, the results also show that older adults need much more time to achieve the same learning outcomes, would welcome frequent repetition and also more time on solving individual tasks; simply, the design and execution of the whole course should be tailored to their specific needs as pointed out by Pappas et al. (2019). Nevertheless, the results did not show any improvement in seniors’ wellbeing (Ware et al., 2017; van den Berg, 2019). This might have been caused either by the short duration of these courses or by the computerized aspect of FLL, which does not provide much space for socializing when learning individually. However, as Formosa (2019) states, maintaining cognitive skills and expanding social contacts are the most common reasons for attending such a course. Klimova and Pikhart (2020) report that it is especially through social and psychological wellbeing that the cognitive benefits of FLL might be observed, since FLL can be for seniors another new purpose of their life. Nevertheless, research also shows that although older adults seem to be highly motivated in conducting meaningful tasks, high motivation is not sufficient for their better performance (Ceccato et al., 2019).

**FIGURE 1:**
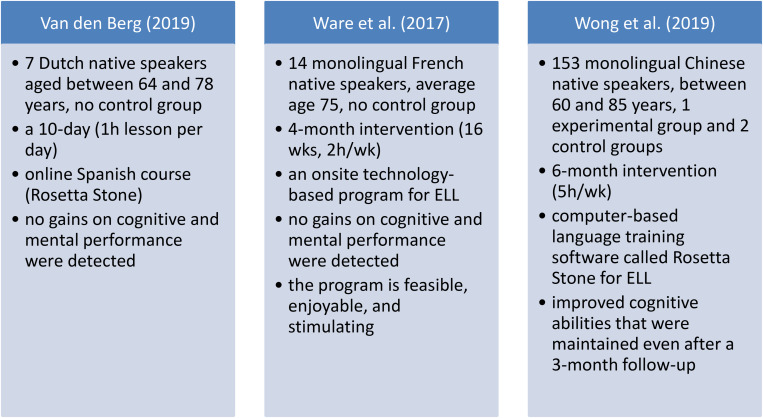
An overview of the key findings of the detected three research studies on the effect of computer-based foreign language training on cognitive benefits among healthy older individuals.

Figure 1 below then summarizes the key findings of the detected three research studies on the effect of computer-based foreign language training on cognitive benefits among healthy older individuals.

Explanations: ELL – English language learning, h – hour, wk – week.

## Conclusion

The findings of this mini-review reveal that the research on cognitive benefits of computer-based foreign language training for healthy older individuals is small-scale. The limited research findings indicate that these computer-based foreign language training programs may bring cognitive benefits for healthy elderly people, especially enhancement of cognitive functions such as working memory. In addition, the authors of the described studies suggest that FLL is a useful activity for healthy older adults since it has the benefits of being meaningful (an advantage over other cognitive training approaches) and provides the chance for acquiring important skills that can enable other aspects of life, such as travel and communication.

The findings of this mini-review contribute to the research on age-related cognitive decline and may be especially of interest to researchers, policy makers, teachers, and learners themselves in the sense that FLL may be a cognitive-reserve-building activity that may give comparative advantage over other cognitive stimulating activities, such as learning to play a music instrument. Language teachers of older adults might be also inspired by pedagogical implications such as incorporating visual-semantic material in their teaching (van den Berg, 2019; Wong et al., 2019), pair classroom learning with individual computerized learning to boost the benefits of social exchange and to maintain learners’ motivation (Ware et al., 2017; van den Berg, 2019) or repeating material frequently (Ware et al., 2017; van den Berg, 2019). It is also desirable to have groups of learners of the same age and language level to avoid feelings of inferiority imposed on them by younger or more proficient learners (Ware et al., 2017).

Future research should focus on more rigidly and methodologically designed randomized controlled studies, particularly on those in which one group would be trained only in FLL and the other would learn a foreign language via computer in order to detect whether there are any differences in effect sizes, as well as identifying differences in wellbeing gains among both groups.

## Author Contributions

The author drafted, analyzed, wrote, and read the entire manuscript.

## Conflict of Interest

The author declares that the research was conducted in the absence of any commercial or financial relationships that could be construed as a potential conflict of interest.
